# Beauty and the pathogens: A leaf-less control presents a better image of Cymbidium orchids defense strategy

**DOI:** 10.3389/fpls.2022.1001427

**Published:** 2022-09-13

**Authors:** Sagheer Ahmad, Guizhen Chen, Jie Huang, Kang Yang, Yang Hao, Yuzhen Zhou, Kai Zhao, Siren Lan, Zhongjian Liu, Donghui Peng

**Affiliations:** ^1^Key Laboratory of National Forestry and Grassland Administration for Orchid Conservation and Utilization at College of Landscape Architecture, Fujian Agriculture and Forestry University, Fuzhou, China; ^2^College of Life Sciences, Fujian Normal University, Fuzhou, China

**Keywords:** leaf-less control, orchids, pathogens, biological control, immunity, defense

## Abstract

Biological control is a safe way of combating plant diseases using the living organisms. For the precise use of microbial biological control agents, the genetic information on the hypersensitive response (HR), and defense-related gene induction pathways of plants are necessary. Orchids are the most prominent stakeholders of floriculture industry, and owing to their long-awaited flowering pattern, disease control is imperative to allow healthy vegetative growth that spans more than 2 years in most of the orchids. We observed leaf-less flowering in three orchid species (*Cymbidium ensifolium*, *C. goeringii* and *C. sinense*). Using these materials as reference, we performed transcriptome profiling for healthy leaves from non-infected plants to identify genes specifically involved in plant-pathogen interaction pathway. For this pathway, a total of 253 differentially expressed genes (DEGs) were identified in *C. ensifolium*, 189 DEGs were identified in *C. goeringii* and 119 DEGs were found in *C. sinense*. These DEGs were mainly related to bacterial secretion systems, FLS2, CNGCs and EFR, regulating HR, stomatal closure and defense-related gene induction. FLS2 (LRR receptor-like serine/threonine kinase) contained the highest number of DEGs among three orchid species, followed by calmodulin. Highly upregulated gene sets were found in *C. sinense* as compared to other species. The great deal of DEGs, mainly the FLS2 and EFR families, related to defense and immunity responses can effectively direct the future of biological control of diseases for orchids.

## Introduction

Plants have developed a multi-layered defense system to resist the invasion of pathogens (Kaur et al., 2022). In the primary response, plants recognize pathogens by using PTI (PAMP-triggered immunity) which uses cell-surface pattern-recognition receptors (PRRs). Defense-related genes are activated to produce antimicrobial compounds through MAPK signaling pathway triggered by FLS2 and EFR (Nishad et al., 2020). The increase in the concentration of Ca^2+^ in the cytosol regulates the production of ROS (reactive oxygen species) and HR (hypersensitive response)/programmed cell death. The secondary response is called ETI (effector-triggered immunity). In this response, pathogens use secretion systems to inject effector proteins into the plant cells to suppress PTI. Pathogens also invade the immunity of the host plants by manipulating their hormone signaling pathways. In response to pathogen effectors, plants use intracellular surveillance proteins called R proteins to monitor the presence of the virulence proteins from pathogens. The ETI arrests pathogen growth by localized programmed cell death, which causes cultivar-specific disease resistance (Nishad et al., 2020).

FLAGELLIN SENSING 2 (FLS2) is an important regulator of plant immunity against pathogenic bacteria (Zipfel et al., 2004). It acts as a PRR (pattern recognition receptor) for flagellin, which is the building block of bacterial flagellum and is perceived as PAMP (pathogen-associated molecular pattern) protein in animals and plants (Gómez-Gómez and Boller, 2000; Hayashi et al., 2001). PRRs proteins contain single transmembrane bearing N-terminal receptor-like domain that recognizes ligands. FLS2 is the first PRR identified in plants and belongs to a large family of receptor kinases (Robatzek and Wirthmueller, 2013). It can recognize a conserved 22-amino acid peptide called flg22 in the N-terminus of flagellin (Felix et al., 1999; Bauer et al., 2001; Chinchilla et al., 2006). In Arabidopsis, other PRRs include EFR (EF-Tu receptor) that identifies the elf18 peptide corresponding to the N-terminus of the bacterial EF-Tu, and CERK1 (chitin elicitor receptor kinase 1) that recognizes the chitin of fungi and the bacterial peptidoglycans (Zipfel et al., 2006; Miya et al., 2007; Petutschnig et al., 2010; Shafique et al., 2011; Willmann et al., 2011). In rice, chitin-elicitor-binding protein, a receptor protein, functions together with *OsCERK1* for chitin perception (Shimizu et al., 2010). The receptor kinase XA21 uses the XA21 peptide sensing to create resistance against *Xanthomonas oryzae* bacteria (Lee et al., 2009). Tomato receptor proteins LeEix1/2 confer the perception of xylanase from fungi in the ethylene-induction pathway (Ron and Avni, 2004). Moreover, PRRs can perform heterologous functions (Monaghan and Zipfel, 2012). For example, *Ve1* is a PRR in tomato and mediates immunity against *Verticillium* fungus, while it can also provide resistance against *Verticillium* in Arabidopsis (Fradin et al., 2011; De Jonge et al., 2012).

FLS2 regulates the ROS (reactive oxygen species) production, the defense-related hormones (salicylic acid and ethylene), deposition of secondary compounds (e.g., callose) and the transcriptional reprogramming involving WRKY TFs (Boller and Felix, 2009), thereby establishing plant immunity. FLS2 makes a stable complex with BRI-associated kinase 1 (BAK1) in a flg22-dependent manner (Robatzek and Wirthmueller, 2013). BAK1 is a regulatory transmembrane receptor kinase and a member of SERK (somatic embryo receptor kinase) family; it involves brassinosteroid signaling and flg22-activated multiple immune responses (Li et al., 2002; Chinchilla et al., 2007; Heese et al., 2007; Roux et al., 2011; Schwessinger et al., 2011). BAK1 and FLS2 interact with BIK1 (botrytis-induced kinase 1) and its homologs (PBL1, PBL2, and PBS1); while BIK1 is receptor-like cytoplasmic kinase (Robatzek and Wirthmueller, 2013). MAPK (mitogen-activated protein kinase) cascades work downstream of FLS2-BAK1 complex in response to flg22 (Asai et al., 2002; Ichimura et al., 2006). Moreover, FLS2-BAK1 dimerization activates CDPK (calcium dependent protein kinase) signaling pathways (Boudsocq et al., 2010).

Calcium signaling plays important roles in symbiotic and pathogenic plant-microbe interactions (Zipfel and Oldroyd, 2017; Mubeen et al., 2022; Rhodes et al., 2022). PRRs trigger a transiently rapid influx in the cytoplasmic calcium, depending upon the PRR complex and downstream RLCKs (receptor-like cytoplasmic kinases; Ranf et al., 2012, 2014; Li et al., 2014; Monaghan et al., 2015; Ahmad et al., 2018). Recently, a number of calcium channels have been implicated, such as CNGCs (cyclic nucleotide-gated channels; Rhodes et al., 2022). The NRL-triggered cell death, known as HR (hypersensitive response), depends on calcium (Bashir et al., 2013). Treatment with calcium channel blockers and calcium chelators can inhibit cell death (Levine et al., 1996; Grant et al., 2000; Ali et al., 2007).

Perception of PAMP by PRRs instigates a defense response called PTI (pattern-triggered immunity) to inhibit pathogenic infections (Macho and Zipfel, 2014). Against it, pathogens use effector molecules to suppress PTI (Wang et al., 2022) and plants have evolved intracellular receptors to counter effector molecules. These receptors are called NLR (nucleotide-binding, leucine-rich repeats) proteins which activate ETI (effector-triggered immunity; Ngou et al., 2022). PTI and ETI have been considered as two independent pathway layers regulating plant immune system (Rhodes et al., 2022). PRR activation also triggers the phosphorylation of MAPK cascades. MAP kinase kinase kinase (MAPKKK/MEKK) activation triggers the downstream MAP kinase kinase (MAPKK/MKK), which stimulates the downstream MAPK/MPK (Asai et al., 2002; Ichimura et al., 2002; Akram et al., 2014; He et al., 2018; Komis et al., 2018).

The Orchidaceae falls among the largest and the highly evolved monocot plant families (Roberts and Dixon, 2008). Approximately, 70,000 orchid species have been cultivated worldwide as medicinal and ornamental plants (Wong, 2002). The Cymbidium orchids are best known for their ideal characteristics and aesthetic appeal around the world (Cribb, 2014). However, there are a number of biotic (viral, bacterial and fungal diseases) and abiotic (salinity and drought stress) factors that seriously affect the quality and production of orchids (Tuhid et al., 2012; Akram et al., 2019; Ren et al., 2020). For example, Tobamovirus and CymMV (Cymbidium mosaic virus) are the lethal viral pathogens causing necrosis, chlorosis and dwarfism in orchids, which causes huge ornamental and economic losses to orchids (Koh et al., 2014). A little information is available on the immunity mechanism of orchids, although their long vegetative phase requires a strong defense strategy to cope with pathogens. Such studies indicate the orchid immunity and defense responses for some specific diseases. A transcriptome-wide analysis for multiple orchid species has not been described, especially the Cymbidium orchids. Moreover, a leaf-less control has not been discussed before, that can produce much more genetic information on immunity regulation in the healthy leaves. For an effective biological control, the understanding of orchids defense machinery is a demanding area of research. Therefore, this study identifies an ideal leaf-less control to study the genetic makeup of immunity responses for Cymbidium orchids. Three ideal Cymbidium orchids, *C. ensifolium*, *C. goeringii* and *C. sinense* were used to perform reference-based transcriptome analysis and then genes related to plant-pathogen interaction pathway and MAPK-signaling pathways were ascertained. This study provides a broader image of orchid defense mechanism and maximizes the immunity information than ever before. The outcomes, thus, suggest the effective immunity mechanism to devise biological control strategy for orchids and other floriculture crops.

## Materials and methods

### Induction of leafless protocorm growth

Three orchid species (*C. ensifolium*, *C. goeringii*, and *C. sinense*) were grown through protocorms in long jars with special media containing 6-BA (8.0 mg L^−1^), NAA (0.5 mg L^−1^), sugar (35 g L^−1^), activated carbon (1.5 g L^−1^) and agar (7.0 g L^−1^). The chamber temperature was set to 26 ± 2 ^o^C with a photoperiod of 12 h/day and light intensity of 2,500–3,000 Lx.

Two types of protocorms were obtained for each species; at an age of 6 months, the leaf-less plants which produced flowers without vegetative growth and the normal plants with leaf and root growth. Three replicates were obtained for each pattern of each species for RNA Sequencing.

### RNA-seq library preparation and sequencing

A total of 18 (6 tissues in 3 replicates) tissues were obtained from 6 month old plants to extract RNA suing the TaKaRa kit. The total RNA was used to prepare cDNA libraries. The mRNA was obtained with Oligotex Midi Kit for mRNA (Qiagen, Germany) and the quality assessment was done with Nano-Drop spectrophotometer (Thermo Fisher Scientific, United States). The cDNA libraries were prepared by using Illumina protocol. The library product evaluation was made through Agilent 2,200 TapeStation and Qubit®2.0 (Life Technologies, United States), followed by product dilution to 10 pM to generate *in situ* clusters on HiSeq2500 pair-end flow cells and pair-end sequencing (2 × 100). Finally, the reference-based transcriptome sequencing was performed using the reference genomes of each species. Gene expression was measures as FPKM (fragments per kilobase per transcript per million mapped reads).

### Functional annotation

The assembled genes were mapped to publically available databases, such as NR (non-redundant), GO (Gene Ontology), KEGG (Kyoto Encyclopedia of Genes and Genomes) and KO (KEGG ortholog) databases using the BLASTX program with a threshold E-value ≤10-5. The GO and KEGG annotations were further classified into functional categories and pathways using the phyper function in R software. The corrected *p*-value was obtained by FDR and the terms with functional *Q*-value ≤0.05 were considered as significantly enriched (Ahmad et al., 2021).

### Differentially expressed genes

The Bowtie2 program (v2.4.4, Johns Hopkins University) was used to align clean reads to genomic sequences and the expression level of each sample was calculated using RSEM (v1.2.8) with default parameters. Then the DEGseq package (1.10.1) was used in R software to obtain DEGs. The DEGs were sorted at a threshold value of *p* < 0.001 and the log2FC > 1 (Ahmad et al., 2022).

### Plant defense related gene identification

From the DEGs, we filtered the genes related to two key pathways that play important roles in the regulation of defense responses and plant immunity against pathogens. These pathways included Plant-pathogen interaction pathway (ko04626) and MAPK signaling pathway (ko04016). The genes for each pathway were divided into different categories according to their up- and down-regulated trends.

### Common interaction network for three species

The common gene names were obtained for defense-related genes for three species and their protein sequences were run on online string facility^1^ to generate a network. The network annotation was shown as GO biological processes for all the genes.

### Highly upregulated and downregulated genes and pathogen stress pathway

The top five highly upregulated and highly downregulated genes were identified for each species in plant-pathogen interaction pathway. Their expression intensities were drawn as heatmap using TBtools.^2^ Finally, the pathway integrators were shown for pathogen stress in a combined form for three species.

## Results

### Expression analysis and pairwise comparison of DEGs

**Figure 1:**
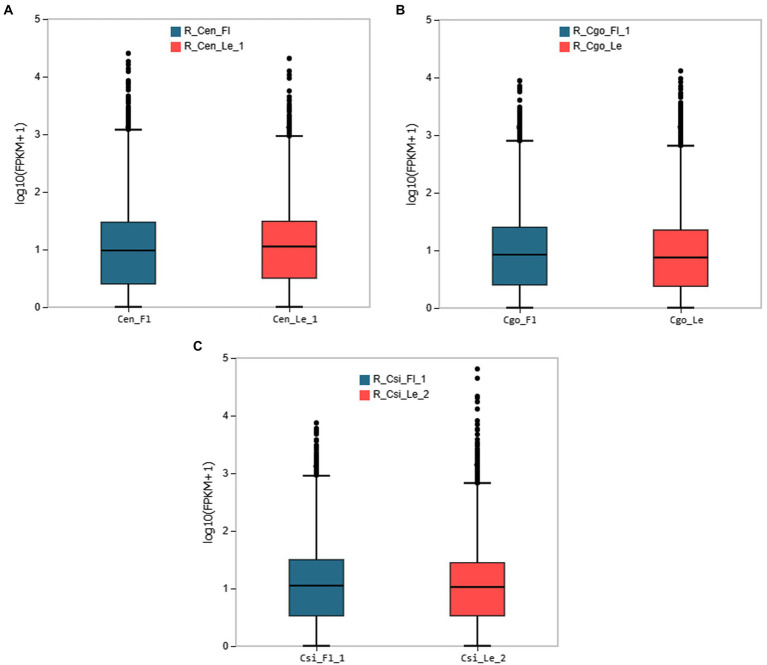
The boxplot distribution of gene expression from the two tissues of each species; **(A)** *C. ensifolium*, **(B)** *C. goeringii*, **(C)** *C. sinense*. The X-axis shows the sample name; the Y-axis represents log10(FPKM+1). The boxplot of each area shows five statistics (from top to bottom are the upper limit, upper quartile, median, and lower quartile, respectively).

The expression patterns for leaf and leaf-less flowers were observed for each species using the empirical cutoff values of genes with positive expressions. The boxplots in Figure 1 shows the expression distribution of the FPKM values of genes. The boxplots curtail the uniform distribution of median and quartile values of DEGs among samples of each species.

**Figure 2:**
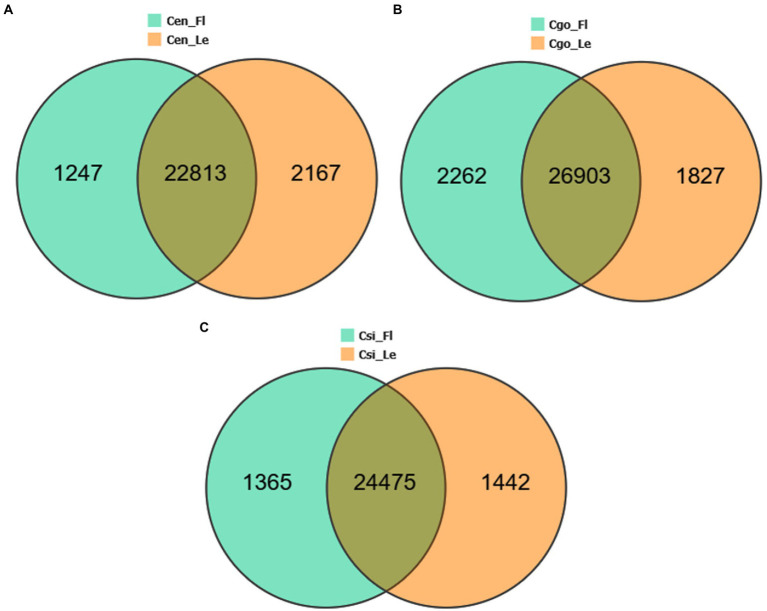
Individual species tissue-specific and common DEGs among the leaf-less control and leaf samples of *C. ensifolium* **(A)**, *C. goeringii* **(B)**, and *C. sinense* **(C)**.

The DEGs were compared between leaf-less flowers and leaf samples within each species separately (Figure 2). The highest number of up- and down-regulated genes (4089) can be seen for *C. goeringii* (Figure 2B), followed by *C. ensifolium* (3414; Figure 2A) and *C. sinense* (2807; Figure 2C). *C. ensifolium* showed the highest number of leaf-specific upregulated genes (2167) as compared to *C. goeringii* (1827) and *C. sinense* (1442). Interestingly, the highest numbers of downregulated genes were observed in the leaves of *C. goeringii* (2262), which was significantly different from *C. ensifolium* (1247) and *C. sinense* (1365).

### GO and KEGG annotation analysis

**Figure 3:**
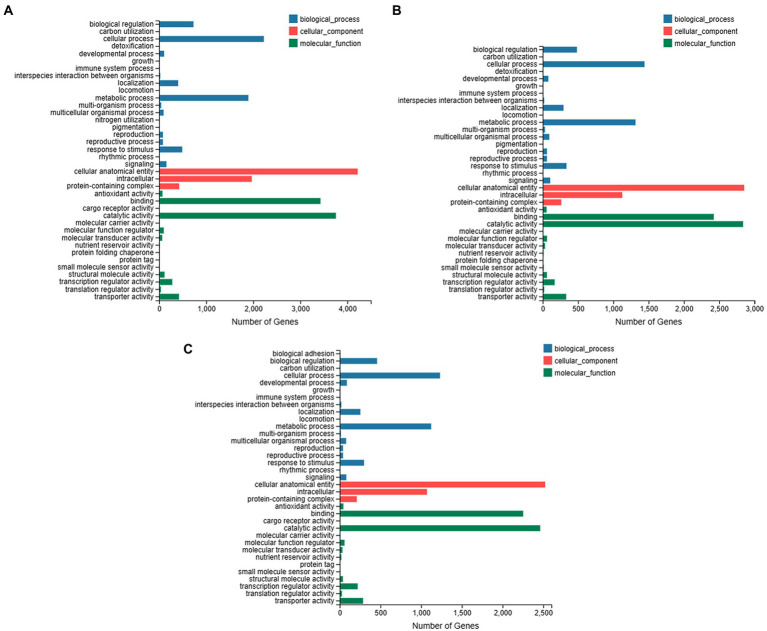
Overview of GO enrichments for *C. ensifolium* **(A)**, *C. goeringii* **(B)**, and *C. sinense* **(C)**. The DEGs enrichment is shown in three GO categories, including biological process, cellular component and molecular function. The y-axis shows the GO category and the x-axis shows the number of genes enriched to each category.

The GO annotation was obtained for individual species (Figure 3). For *C. ensifolium* the maximum number of genes was related to cellular process and metabolic process in the GO biological process category (Figure 3A). For the cellular component category, the highest number of genes was enriched for cellular anatomical entities followed by intracellular components. The major molecular functions were related to catalytic activity and binding (Figure 3A). In the case of *C. goeringii* (Figure 3B), the GO annotations were similar to *C. ensifolium*. Although similar GO enrichments were observed for *C. sinense* (Figure 3C), however, the number of genes were less than other two species.

**Figure 4:**
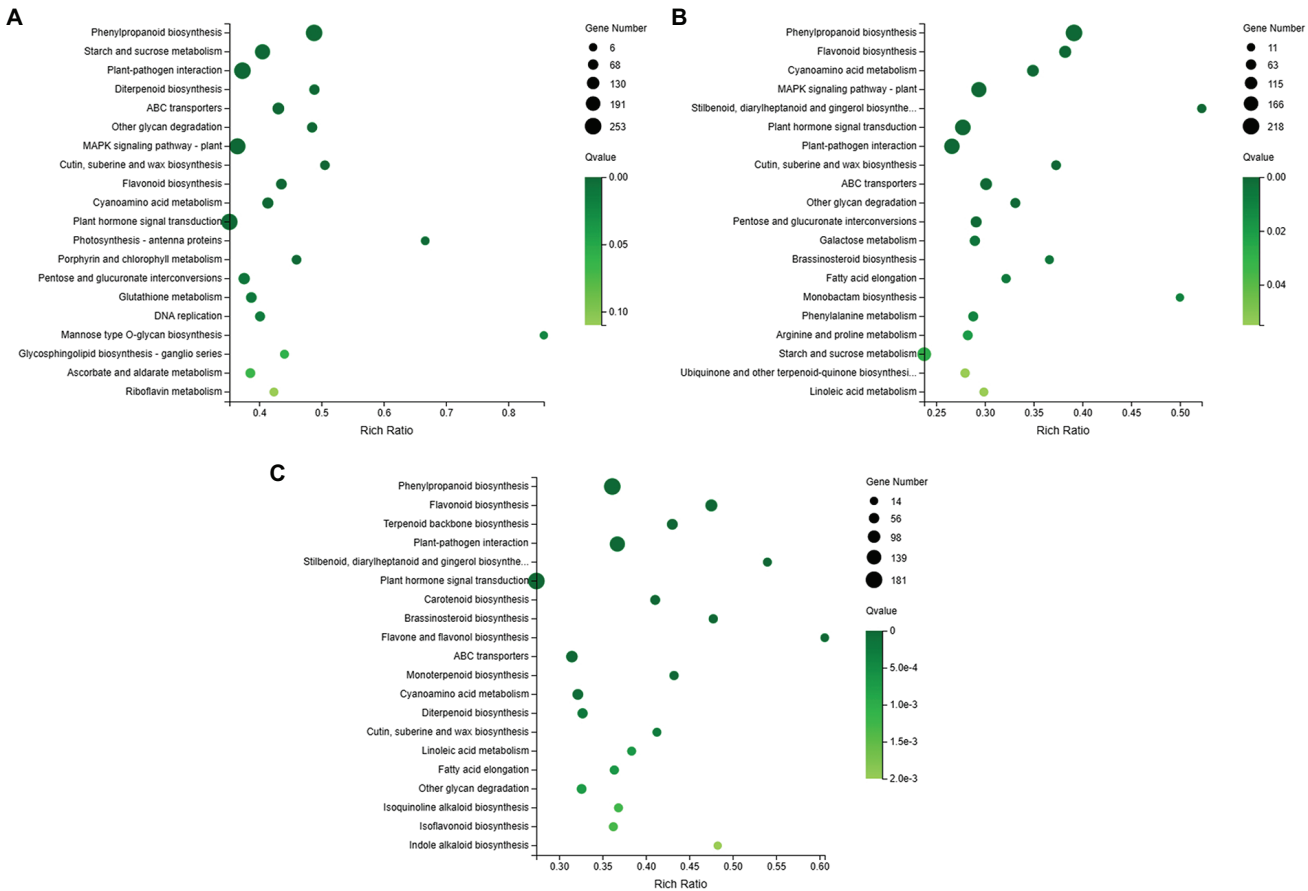
Overview of KEGG enrichments for *C. ensifolium* **(A)**, *C. goeringii* **(B)**, and *C. sinense* **(C)**. The y-axis shows the KEGG pathway names and the x-axis shows the pathway-richness ratio. The size of the black circles shows the number of genes enriched to each pathway; bigger the circle size more is the number of genes.

The plant-pathogen interaction pathway (ko04626) was counted among the most enriched KEGG pathways in the three orchid species (Figure 4). Highly enriched number of genes for this pathway were found in *C. ensifolium* (Figure 4A), followed by *C. goeringii* (Figure 4B) and *C. sinense* (Figure 4C). MAPK signaling pathway was prominent among the other pathways in *C. ensifolium* and *C. goeringii*, however, it was not prominent in *C. sinense*. The other major pathways with significant gene enrichment included plant hormone signal transduction, phenylpropanoid biosynthesis, and starch and sucrose metabolism, which may indirectly play roles in plant defense and immunity responses.

### Defense responses for each species

**Figure 5:**
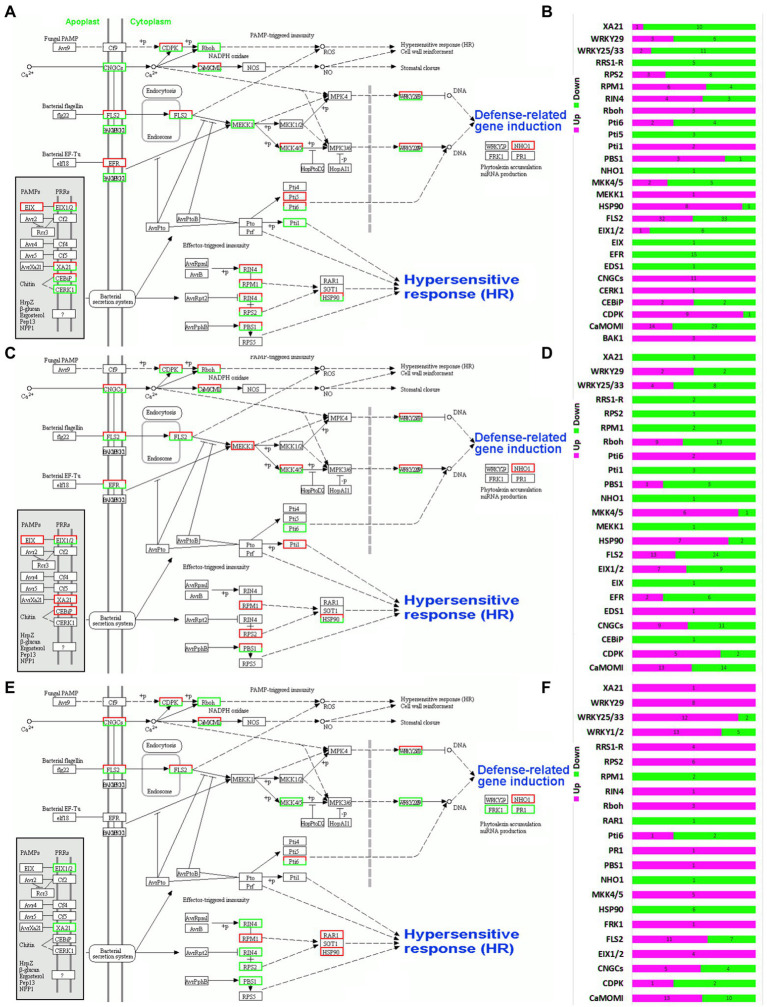
Overview of defense response pathway and the distribution of up- and downregulated pathway integrators for *C. ensifolium* **(A,B)**, *C. goeringii* **(C,D)**, and *C. sinense* **(E,F)**.

A number of plant-pathogen interaction response pathways were observed in the three orchid species (Figure 5). All the routes were converged to the three key responses, i.e., defense related gene induction, stomatal closure and hypersensitive response. The stimulation originates from the cell membrane and the final responses are shown in the cytoplasm of plant cells. Four key response generators were observed in the three species, including CNGCs, FLS2, EFR and bacterial secretion system (Supplementary Tables 1–3). In *C. ensifolium*, the genes related to CNGCs were upregulated in leaves as compared to leaf-less flowers (Figure 5A). Contrarily, the EFR related genes were downregulated in the leaves. The FLS2 contained both upregulated and downregulated genes. The other solely upregulated genes were related to Rboh in the CNGCs routes, MEKK1 in the FLS2 route and *Pti1* in the bacterial secretion route. All other regulators contained both upregulated and downregulated genes (Figure 5B). Higher number of downregulated genes were observed in *C. ensifolium* leaves than upregulated genes among the 27 expressed pathway stimulators (Figure 5B). Moreover, *NHO1* was the only defense-related gene downregulated in the leaves of three species.

Contrary to *C. ensifolium* the EFR contained both upregulated and downregulated genes in *C. goeringii* (Figure 5C). Moreover, the *MEKK1* and *Pti1* were completely downregulated, while Pti6 was upregulated as compared to *C. ensifolium*. In the bacterial secretion route, the *RPM1* and *RPS2* were completed downregulated in the *C. goeringii* leaves as compared to *C. ensifolium* (Figure 5C). Similar to *C. ensifolium*, *NHO1* was the only defense related gene downregulated in the leaves of *C. goeringii*. Moreover, the number of downregulated genes was higher than the upregulated genes (Figure 5D).

The defense-related genetic map of *C. sinense* was much different than other two species (Figures 5E,F). In the CNGCs rout, Rboh genes were completely upregulated in the leaves. In the FLS2 route, *MKK4/5* and *WRKY22* and *WRKY29* were also upregulated. In the bacterial secretion route, the *RIN4*, *RPS2* and *PBS1* were upregulated, while, the *RAR1* and *HSP90* were completely downregulated. Three defense-related genes were induced in *C. sinense* as compared to other two species. Here, the *FRK1* and *PR1* were upregulate and *NHO1* was downregulated. Most of the genes were upregulated in *C. sinense* contrary to other two species, while a few were downregulated (Figure 5F).

### Interaction networks by defense proteins

**Figure 6:**
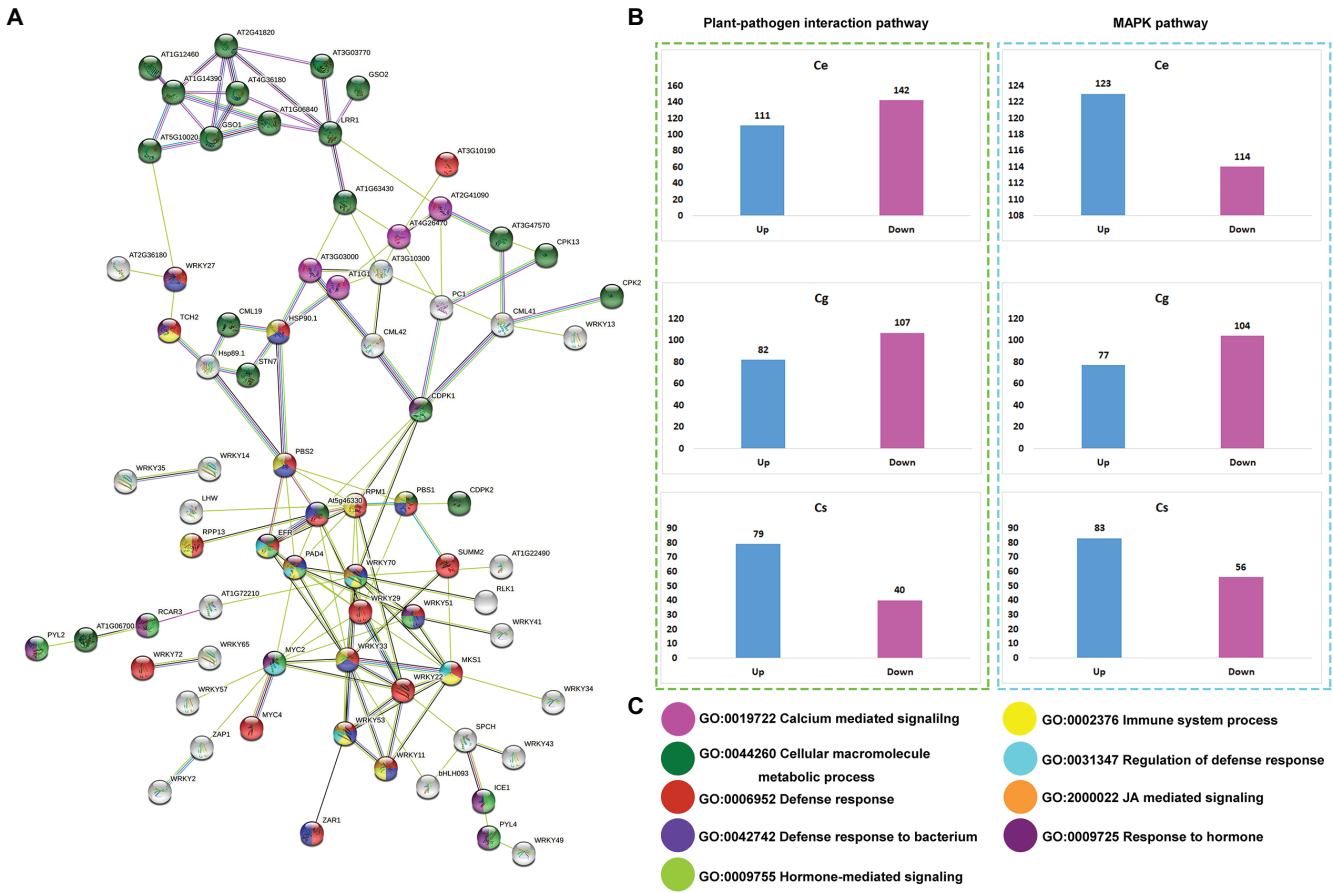
Identification string-based interaction network for all the genes expressed in three orchid species **(A)**, up- and downregulated genes for plant-pathogen interaction pathway and MAPK signaling pathway for three orchid species **(B)**, and the representation of major biological process enriched in the interaction network **(C)**.

The protein sequences of plant-pathogen interaction and MAPK signaling pathway genes were run on the online string database to see their interaction patterns (Figure 6A). All the important regulators of plant defense and immunity were interconnected. The interconnected proteins were mainly involved in calcium mediated signaling (GO:0019722), cellular macromolecule metabolic process (GO:0044260), defense responses (GO:0006952), defense response to bacteria (GO:0042742), hormone-mediated signaling (GO:0009755), immune system process (GO:0002376), and JA-mediated signaling (GO:2000022; Figure 6C).

Up-and down-regulation profiles were compared for both the pathways (Figure 6B). For plant-pathogen interaction pathway the number of downregulated genes was higher than the number of upregulated genes in *C. ensifolium* and *C. goeringii* as compared to *C. sinense*, where the number of downregulated genes (40) was significantly lower than the number of upregulated genes (79; Figure 6B). For MAPK signaling pathway, the number of upregulated genes was higher than that of downregulated genes in *C. ensifolium* and *C. sinense*. However, the number of upregulated genes (77) was significantly lower than the number of downregulated genes (104) in *C. goeringii*.

### Highly tissue-specific gene sets

**Figure 7:**
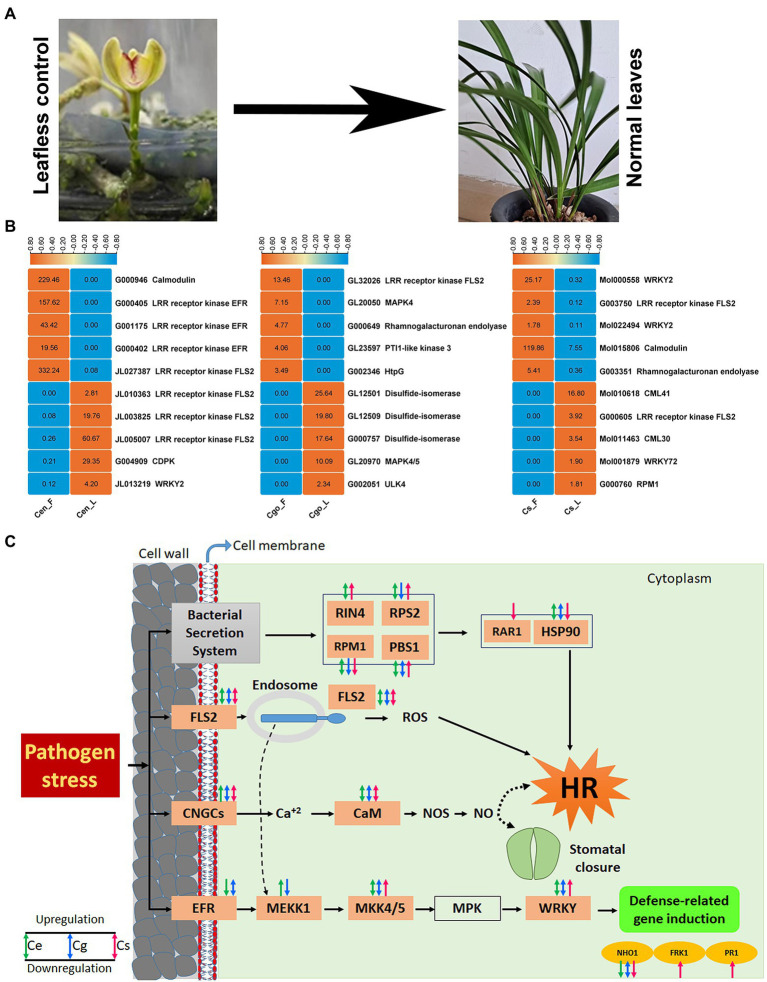
Leaf-less control and the healthy leaf samples used for sequencing analysis **(A)**, top 5 highly upregulated and highly downregulated defense-related DEGs **(B)**, and the final pathway of pathogen stress showed by three orchid species **(C)**. The orange boxes show the genes expressed in either of the species.

The leaf-less controls provides the best control to study the defense and immunity mechanism in the leaf (Figure 7A). Using this control, we found the top 5 highly upregulated and highly downregulated genes in the leaves of three orchid species (Figure 7B). In *C. ensifolium*, the downregulated genes included calmodulin, three EFRs and one FLS2, while the upregulated genes included three FLS2s, *CDPK* and *WRKY2*. In *C. goeringii*, the downregulated genes included FLS2, MAPK4, rhamnogalacturonan endolyase, Pti1-like and HtpG, while the upregulated genes included three disulfide isomerases, MAPK4/5 and ULK4. In the case of *C. sinense*, the downregulated genes included two *WRKY2*, FLS2, calmodulin and rhamnogalacturonan endolyase, while the upregulated genes were *CML41*, *FLS2*, *CML30*, *WRKY72*, and *RPM1* (Figure 7B).

In the orchid species, the defense-related responses are induced by four key routes, including bacterial secretion system, FLS2, CNCGs, and EFR (Figure 7C). The bacterial secretion system stimulates four proteins in orchids, such as RIN4, RPS2, RPM1 and PBS1. RIN4 was not expressed in *C. goeringii*, while the remaining three were expressed in all the three species. These proteins ultimately instigate hypersensitive response (HR) through the stimulation of *RAR1* and *HSP90*. *RAR1* was only downregulated in *C. sinense*, while it was not expressed in other two species. *HSP90* was downregulated in *C. sinense* and showed both up- and down-regulation in *C. ensifolium* and *C. goeringii*. FLS2 also triggers HR through reactive oxygen species (ROS). Both up- and downregulation profiles were found for FLS2 in the three orchid species. The CNGCs use calcium signaling to induce CaM, which then cause HR and stomatal closure through NOS and NO signals. Both up- and downregulation profiles were found for CNGCs and CaM in the three orchid species.

The EFR routes works differently than the previous three routes and cause the induction of defense-related genes. It involves multiple regulatory before the final response. These regulators include *MEKK1*, *MKK4/5*, *MPK* and *WRKY*, which finally induce defense-related genes, such as *NHO1*, *FRK1* and *PR1*. EFR and MEKK1 genes were not expressed in *C. sinense*, while MPK genes were not expressed in any of the species. The *MKK4/5* and *WRKY* were expressed in all the species. Among the defense-related genes, the *NHO1* was expressed in all the species, while the *FRK1* and *PR1* were expressed only in *C. sinense* (Figure 7C).

## Discussion

Orchids share a huge portion of floriculture industry. Obtaining virus free orchids through meristem culturing has been used to control the spread of diseases (Panattoni et al., 2013). However, tissue culturing of orchids is very costly and time-consuming and their long vegetative phase (2–3 years) requires a permanent solution against pathogens. The mechanisms of plant defense and immunity against pathogens have been documented in numerous model plants. However, limited genomic information has been presented on the orchids, especially the Cymbidium orchids. A detailed and genome-wide searching of defense-related genes would facilitate the future breeding programs for disease-resistance in Orchidaceae.

Reference-based transcriptome sequencing produced significantly upregulated and downregulated gene profiles for leaf-less control and leaf samples for each of three orchid species, *C. ensifolium*, *C. goeringii* and *C. sinense* (Figure 2). Cellular and metabolic process were the most enriched GO biological processes, mainly involving catalytic and binding activities in the cellular anatomical entities (Figure 3). Plant-pathogen interaction and MAPK signaling pathways were highly enriched KEGG terms in the three orchid species (Figure 4) among the other pathways including, mainly, plant hormone signal transduction and phenylpropanoid biosynthesis pathways. The data enrichment suggests the significance of plant defense and immunity responses of orchids.

Highly upregulated and downregulated transcripts associated with plant-pathogen interaction were induced for the leaves of three orchid species. For example, LRR receptor kinases encoding transcripts were highly expressed in the leaves as compared to leaf-less controls. EFR and FLS2, LRR receptor-like kinases, are the primary response elements and act to recognize bacterial epitopes elf18 and flg22 (Figures 5A,C,E; Saijo et al., 2009; Ahmad et al., 2014). A total of 65 FLS2-related transcripts (32 upregulated and 33 downregulated) were found in the leaves of *C. ensifolium* (Figure 5D), while 15 EFRs were found, which were all downregulated. In *C. goeringii*, 37 FLS2s were found (13 upregulated and 24 downregulated; Figure 5F), while there were 8 EFRs (2 upregulated and 6 downregulated). *C. sinense* contained 18 FLS2s (11 upregulated and 7 downregulated) and no EFR expressed here (Figure 5F).

CML and CaM are calcium sensors and play pivotal roles in calmodulin signaling pathways involving oxidative burst and cell death regulation in the infected plants enduring HR (Harding et al., 1997; Harding and Roberts, 1998; Poovaiah et al., 2013). A total of 43 CaM transcripts were identified in *C. ensifolium* (14 upregulated and 29 downregulated; Figure 5B), 27 in *C. goeringii* (13 upregulated and 14 downregulated; Figure 5D), and 23 CaMs were identified in *C. sinense* (13 upregulated and 10 downregulated; Figure 5F).

Plants use specific pattern recognizing receptors to identify microbes. These receptors are activated by MAMPs (microbe associated molecular patterns), which results into MTI (MAMP-triggered immunity). However, clever pathogens bypass MTI using virulence effectors, thereby causing pathogen proliferation. In the resistant genotypes, intracellular immune receptors detect these effectors and start defense responses called ETI (effector triggered immunity), including HR and transcriptional reprogramming in order to halt the growth of pathogens. *Pseudomonas syringae* secretes AvrRpm1 effector into the host cell to induce virulence. *RIN4* negatively regulates MTI and its modification in the presence of effectors like ArvRpm1. The *RPM1* and *RPS2* are a nucleotide binding leucine-rich repeat sensors. *RPM1* perceives the perturbation of *RIN4* in disease resistance, which also activates the *RPS2*, causing an enhanced immune response (Cherkis et al., 2012). Heat shock proteins (HSP90) involve detoxification and stress responses (Azaiez et al., 2009). The AvrB effector of *P. syringae* suppresses PTI by using RAR1, an HSP90 cochaperone required for ETI, indicating that RAR1 is a negative regulator of PTI (Shang et al., 2006). Our data included all the components responding to ETI, including *RIN4*, *RPS2*, *RPM1*, *PBS1*, *HSP90*, which were expressed in all the species, except *RAR1* which was only expressed but downregulated in the leaves of *C. sinense* (Figure 7C), suggesting the immunity level of Cymbidium orchids against RAR-mediated pathogenic attack.

Among the highly expressed genes, EFRs were downregulated in the leaves of *C. ensifolium*, while FLS2s were upregulated. A number of WRKY transcription factors were differentially expressed, such as *WRKY25*, *WRKY29*, and *WRKY33* (Figures 5B,D,F). WRKY TFs have admitted role in the regulation of plant defense responses (Eulgem, 2006; Knoth et al., 2007; Azaiez et al., 2009; Faiz et al., 2022). *WRKY2* represses the basal immunity of barley by directly targeting PAMP recognition receptor genes (Yu et al., 2022). It was upregulated in the leaves of *C. ensifolium*, while downregulated in the leaves of *C. sinense* (Figure 7), suggesting that orchids may differ in their immunity responses even within the same genus. Similarly FLS2 has also dual roles with both upregulated and downregulated genes in the three species.

Previous studies show that flg22 triggers MAP kinases, such as MEKK1, MKK4/5 and MPK (Nuhse et al., 2000; Asai et al., 2002). Three flg22-inducible genes, including *NHO1*, *FRK1*, and *WRKY29* (An and Mou, 2012), are induced by this cascade of kinases in the EFR pathway. We found up- and downregulated transcripts for *EFR*, *MEKK1*, *MKK4/5*, and defense-related genes, such as *NHO1*, *FRK1*, and *PR1* (Figure 7C). *NHO1* was expressed in all the species, while *FRK1* and *PR1* were upregulated only in *C. sinense*. Our study, thus, presents a general comparison among three orchid species for a transcriptome-wide mining of defense related genes. This information can be used to plant biological control strategies for orchids and floriculture crops.

## Conclusion

This is the first study that uses leaf-less control to mine the defense and immunity related gene profiles in three Cymbidium orchids. A number of upregulated and downregulated genes were identified in four major routes of pathogen stress, including bacterial secretion system, FLS2, CNGCs and EFR. These routes mainly regulate three processes, namely, hypersensitive response (HR), stomata closure and defense-related gene induction. The regulation of immunity response was a little different in *C. sinense* as compared to *C. ensifolium* and *C. goeringii*. For example, two of the defense related genes (*FRK1* and *PR1*) were upregulated only in *C. sinense*, while they did not express in other two species. Overall, the results present a broad picture of defense machinery of Cymbidium orchids, which can be used to ameliorate pathogen affliction through biological control.

## Data availability statement

The transcriptome sequences described in this article have been submitted to The National Genomics Data Center (NGDC, https://ngdc.cncb.ac.cn) under accession number PRJCA009885.

## Author contributions

SA: conceptualization and writing—original draft. GC: data curation and software. JH: investigation. KY: data curation. YH: software. YZ: visualization, investigation, and editing. KZ: data curation and editing. SL: software and editing. ZL: supervision, conceptualization, and funding acquisition. DP: supervision, conceptualization, funding acquisition, and writing—reviewing and editing. All authors contributed to the article and approved the submitted version.

## Funding

This work was supported by The National Natural Science Foundation of China (no. 32071815); The National Key Research and Development Program of China (2019YFD1001000); The National Key Research and Development Program of China (2018YFD1000401); The Innovation and Application Engineering Technology Research Center of Ornamental Plant Germplasm Resources in Fujian Province (115-PTJH16005); and National Natural Science Foundation of China (no. 32101583).

## Conflict of interest

The authors declare that the research was conducted in the absence of any commercial or financial relationships that could be construed as a potential conflict of interest.

## Publisher’s note

All claims expressed in this article are solely those of the authors and do not necessarily represent those of their affiliated organizations, or those of the publisher, the editors and the reviewers. Any product that may be evaluated in this article, or claim that may be made by its manufacturer, is not guaranteed or endorsed by the publisher.

## Footnotes

^1^https://string-db.org/cgi/input?sessionId=HofWDb4VJD8K (Accessed August 21, 2022).

^2^https://github.com/CJ-Chen/TBtools/releases (Accessed August 21, 2022).
